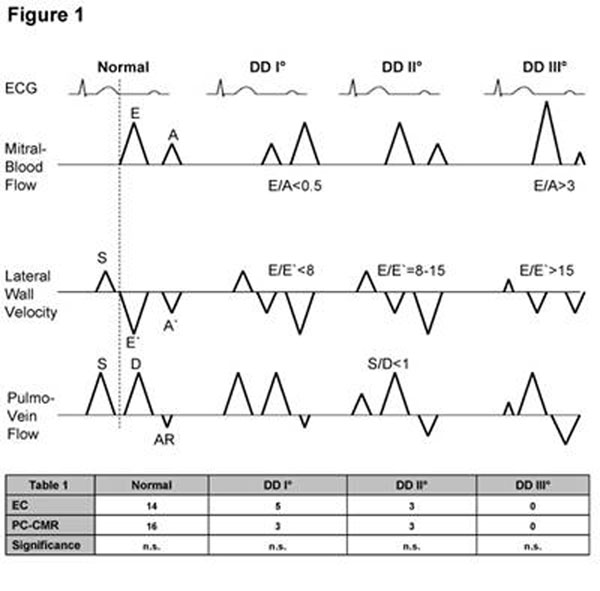# Mri assessment of diastolic dysfunction using the echocardiographic criteria of diastolic mitral blood flow, lateral wall motion velocity and pulmonary vein flow

**DOI:** 10.1186/1532-429X-13-S1-P134

**Published:** 2011-02-02

**Authors:** Liane Kecker, Stephanie Lehrke, Dirk Lossnitzer, Grigorios Korosoglou, Evangelos Giannitsis, Hugo A Katus, Henning Steen

**Affiliations:** 1 University Heidelberg, Heidelberg, Germany

## Introduction

In echocardiography (EC), classification of diastolic dysfunction (DD) is widely accepted and mainly assessed using three criteria (figure1): mitral blood flow (MBF, E-A-curve), lateral wall velocity (LWV, S-E`-A`-curve) and pulmonary vein flow (PVF, S-D-AR-curve). With these three characteristic flow and velocity patterns and their ratios E/A, E/E´ and S/D, regular diastolic function can be clearly distinguished from three degrees of DD (I-III°).

Cardiovascular magnetic resonance (CMR) has excellent capabilities to assess blood flow and myocardial tissue motion using phase contrast (PC-CMR) imaging but has not been used to classify diastolic function similar to the EC approach.

## Purpose

We sought to compare the feasibility of PC-CMR wtih echocardiographic doppler imaging for the assessment of DD using the echocardiographic flow and velocity approach for DD-classification.

## Methods

After acquisition of regular short axis cine SSFP volumetry and 2-3-4chamber views, in 22 patients with various cardiovascular diseases we performed single-slice short-axis PC-CMR (60phases, velocity-encoding=100cm/s) similarly to typical EC locations at the tip of mitral leaflets in diastole on a 1.5T whole body MRI system (Philips Achieva) to generate mitral E-and A-waves, lateral S`-E`-A-velocities, E/A- and E/E`-ratios. PC-CMR for PVF was planned orthogonally to the cine 4-chamber plane 1cm distal from pulmonary vein inflow into the left atrium. Directly after MRI, EC was performed to generate complementary data for MBF, LWV and PVF. After generating all curves and ratios, patients were classified into 4 groups (1=normal;2=DD-I°;3=DD-II°;4=DDIII°) for both techniques.

## Results

EC and PC-CMR could be performed in all patients, whereas EC PVF could not be assessed in 4 patients due to reduced flow signals.

20/22 patients (91%) were categorized similarly, whereas in 2 cases PC-CMR insignificantly underestimated DDI° and mis-diagnosed them as normal. Mean scan-time for MBF, LWV and PVF was 5.20±1.31 min., mean analysis time was 4.10±1.31 min.

## Conclusion

For the first time we could show that PC-CMR analysis of DD is feasible and showed excellent agreement with the widely accepted EC method for classification of DD. PC-CMR could offer the potential of a practically and reasonably time-consuming approach for the clinically important assessment of DD omitting sophisticated MRI sequences and dedicated software analysis tools.

**Figure 1:**